# Identification of Patients Affected by Mitral Valve Prolapse with Severe Regurgitation: A Multivariable Regression Model

**DOI:** 10.1155/2017/6838921

**Published:** 2017-02-02

**Authors:** Paola Songia, Benedetta Porro, Mattia Chiesa, Veronika Myasoedova, Francesco Alamanni, Elena Tremoli, Paolo Poggio

**Affiliations:** ^1^Centro Cardiologico Monzino IRCCS, Milan, Italy; ^2^Dipartimento di Scienze Farmacologiche e Biomolecolari, Università degli Studi di Milano, Milan, Italy; ^3^Dipartimento di Scienze Cliniche e di Comunità, Università degli Studi di Milano, Milan, Italy

## Abstract

*Background*. Mitral valve prolapse (MVP) is the most common cause of severe mitral regurgitation. Besides echocardiography, up to now there are no reliable biomarkers available for the identification of this pathology. We aim to generate a predictive model, based on circulating biomarkers, able to identify MVP patients with the highest accuracy.* Methods*. We analysed 43 patients who underwent mitral valve repair due to MVP and compared to 29 matched controls. We assessed the oxidative stress status measuring the oxidized and the reduced form of glutathione by liquid chromatography-tandem mass spectrometry method. Osteoprotegerin (OPG) plasma levels were measured by an enzyme-linked immunosorbent assay. The combination of these biochemical variables was used to implement several logistic regression models.* Results*. Oxidative stress levels and OPG concentrations were significantly higher in patients compared to control subjects (0.116 ± 0.007 versus 0.053 ± 0.013 and 1748 ± 100.2 versus 1109 ± 45.3 pg/mL, respectively; *p* < 0.0001). The best regression model was able to correctly classify 62 samples out of 72 with accuracy in terms of area under the curve of 0.92.* Conclusions*. To the best of our knowledge, this is the first study to show a strong association between OPG and oxidative stress status in patients affected by MVP with severe regurgitation.

## 1. Introduction

Myxomatous mitral valve prolapse (MVP) is the most common indication for mitral valve surgery due to severe mitral regurgitation (MR). The prevalence of MVP is estimated at 2-3% with approximately 144 million people affected worldwide [1]. Echocardiographically, MVP is defined as a single or bileaflet prolapse, at least 2 mm beyond the long-axis annular plane, while the assessment of valve regurgitation takes into account the effective regurgitant orifice area (EROA) [2]. Interestingly, the pathology is equally distributed between men and women [3] and patients with prolapse have significantly lower body-mass index (BMI) and waist-to-hip ratio than those without prolapse [1].

Despite the pathology was first described in the late 1800s [3], no major risk factor has been identified yet [4] and the triggering mechanisms of MVP are not fully understood. In this context, a very recent study by Deroyer et al. [5] showed a negative association between severity of MR and high-density lipoproteins (HDL) levels. Interestingly, HDL concentrations and levels of Apo-A1, the major protein component of HDL, which participates in the reverse cholesterol transport, decreased according to the severity of MR. In addition, MVP patients showed an alteration in the antioxidant defence systems and an increase in lipid peroxidation markers [6].

Given the background, we investigated Osteoprotegerin (OPG), a well-known protein linked to oxidative stress status [7] and to endothelial mesenchymal transition on endothelial cells isolated from MVP patients [8]. OPG is a secretory glycoprotein of the tumour necrosis factor receptor superfamily involved in calcification, apoptosis, proliferation, and migration processes [9]. It possesses high affinity to receptor activator of nuclear factor-kb (RANKL), to tumour necrosis factor-related apoptosis-inducing ligand (TRAIL), and to syndecan family receptors (SDC). This molecule has been associated with cardiometabolic disorders [9], as well as increased cardiovascular and overall mortality [10, 11]. In addition, it has been shown that transforming growth factor beta (TGF-*β*), a well-known player in myxomatous MVP pathogenesis [12], increases oxidative stress status [13] as well as OPG production and secretion directly stimulating OPG promoter activity [14].

In this study, we aim to generate a predictive model able to identify MVP patients with the highest accuracy with the combination of biochemical parameters easily quantifiable.

## 2. Patients and Methods

### 2.1. Patient Demographics

This observational study was approved by the Institutional Review Board and by the Ethical Committee of Centro Cardiologico Monzino (CCM), IRCCS. The investigation conformed to the principles outlined in the Declaration of Helsinki (1964).

A total of 51 consecutive patients that underwent mitral valve repair, at CCM, due to MVP were enrolled. Based on exclusion criteria (presence of bicuspid aortic valve, premature menopause, and/or osteoporosis, prior aortic or mitral valve surgery, rheumatic heart disease, endocarditis, active malignancy, chronic liver failure, calcium regulation disorders, and chronic or acute inflammatory states), we selected and analysed 43 patients matched by age, sex, diabetes, hypertension, hypercholesterolemia, and smoking habits with 29 control subjects from those attending the clinic for global control of cardiovascular risk at CCM. The demographic and clinical features of the two study groups are listed in Table 1. The subjects were assessed with detailed medical history, physical examination, and echocardiography. In all patients, blood collection was performed before coronary angiography and surgery, with the exception of controls, which underwent samples collection at a scheduled visit.

### 2.2. Blood Sampling and Biochemical Measurements

*Whole Blood*. Peripheral blood sample was drawn from patients and controls while fasting into tubes containing EDTA (9.3 mM; Vacutainer Systems, Becton Dickinson, Franklin Lakes, NJ, USA) kept on ice and immediately precipitated with 10% trichloroacetic acid (Sigma-Aldrich, St. Louis, MO, USA) in 1 mM EDTA solution. After centrifugation at 10,000*g* for 10 min at 4°C, the supernatant was stored at −80°C until analysis.

*Plasma*. EDTA anticoagulated blood was centrifuged at 3,000*g* for 10 min at 4°C within 30 min after being drawn. Plasma was separated and aliquots were stored at −80°C until analysis.

### 2.3. Oxidative Stress Measurement

For oxidative stress evaluation whole blood concentrations of the oxidized (GSSG) and reduced (GSH) form of glutathione, whose ratio (GSSG/GSH) is a well-recognized index, were assessed by a previously developed and validated LC-MS/MS method [15].

### 2.4. Osteoprotegerin Evaluation

Plasma levels of soluble OPG were measured with an enzyme-linked immunosorbent assay (ELISA) kit (DuoSet, R&D) following manufacturer instructions. The standard of this particular kit is similar to full-length OPG, making this ELISA kit more representative of circulating OPG molecule [9].

### 2.5. Statistical Analysis

Continuous variables are summarized as mean ± standard error, except for age, which is represented as mean [minimum, maximum], while categorical variables are summarized as frequency and percentage.

For data analysis, Mann–Whitney test has been performed between MVP and control classes on continuous variables, while Fisher's exact test has been performed on discrete ones.

Finally, taking into account OPG measurement and GSSG/GSH ratio, we have implemented several logistic regression procedures in order to identify a classification rule, able to predict outcomes with the highest accuracy.

The logistic model is

(1) $$\begin{array}{c} p\left(\;Y_{i}=\text{“MVP”}\;\right)=\frac{1}{1+e^{-\left(\beta_{0}+\beta_{1}x_{1}+\beta_{2}x_{2}\right)}}, \end{array}$$

where *p* is probability to have MVP, *x*_1_ is OPG measurement (in pg/mL), and *x*_2_ is GSSG/GSH ratio.

A receiver operating characteristic (ROC) curve has been plotted for each model and performances have been evaluated by comparing areas under the ROC Curve (AUC).

## 3. Results

### 3.1. Patient Characteristics

In this study we analysed 43 patients and 29 controls matched by age, sex, diabetes, hypertension, hypercholesterolemia, and smoking habits. The two groups were comparable for all the clinical and demographic features considered, except for BMI, which was significantly lower in MVP patients than in controls (*p* = 0.005), and as expected, New York Heart Association (NYHA) class that was significantly higher in MVP patients (*p* < 0.001, Table 1). Drug therapies were not significantly different between MVP and controls, apart from beta-blockers, mostly taken by patients. However, up to now, no data are available about any influence of this drug class on oxidative stress levels. In Table 2 the qualitative and quantitative echocardiographic characteristics of mitral valves are reported. As expected the peak* E* velocity and the ratio between peak* E* and peak* A* velocity (*E*/*A* ratio) were significantly different between the two groups (*p* < 0.001).

### 3.2. Osteoprotegerin Levels and Oxidative Stress Status

**Figure 1:**
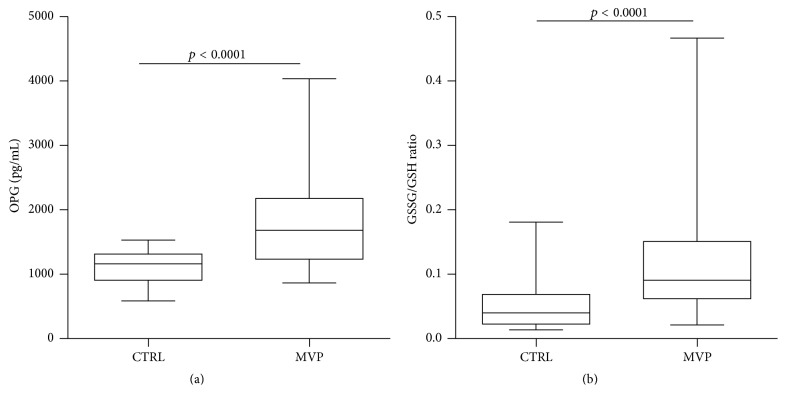
Osteoprotegerin and oxidative stress levels. (a) Osteoprotegerin (OPG) enzyme-linked immunosorbent assay (ELISA) on plasma samples from control subjects (CTRL) and mitral valve prolapse (MVP) patients. (b) Ratio between oxidized (GSSG) and reduced (GSH) form of glutathione as oxidative stress status index.

The assessment of OPG levels revealed that this protein was significantly higher in MVP patients when compared to controls (1748 ± 100.2 versus 1109 ± 45.3 pg/mL, respectively; *p* < 0.0001, Figure 1(a)). Since it has already been shown that OPG concentration increases with age [9], we implemented an adjusted model for this variable and the difference between the groups maintains its significance (*p* < 0.0001). In addition, we adjusted for NYHA class as well as BMI and the difference in OPG levels remained significant between the two groups (*p* < 0.01). Finally, we confirmed that the oxidative stress status, represented by GSSG/GSH ratio, was higher in MVP patients compared to control subjects (0.116 ± 0.007 versus 0.053 ± 0.013, respectively; *p* < 0.0001, Figure 1(b)) and remained significant even after NYHA class and BMI adjustment (*p* < 0.01). Notice that no correlation has been found between NYHA class and OPG levels or GSSG/GSH ratio.

### 3.3. Binary Logistic Regression Model

**Figure 2:**
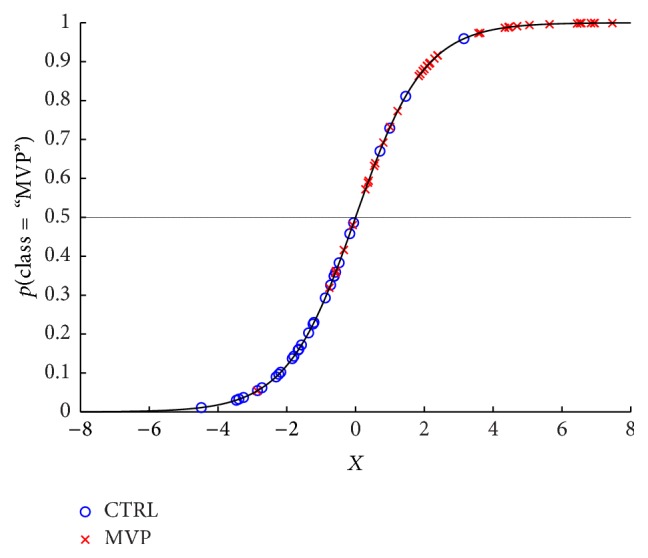
Binary logistic regression model. Graph representing the prediction of the best binary logistic regression model.

To assess if OPG or GSSG/GSH ratio could be used as potential circulating markers of MVP, we implemented a step-wise binary logistic regression model. To further improve the specificity and sensibility, we took OPG and GSSG/GSH ratio alone and then together. We also evaluated their possible interaction; however, it was not statistically significant (*p* = 0.243) and therefore we did not include it in the regression model. The regression parameters obtained from estimation procedure were *β*_0_ = −7.590, *β*_1_ = 0.005, and *β*_2_ = 24.493. The logistic model implemented is described in Section 2.5. This model was able to correctly classify 62 samples out of 72 (Figure 2). In addition, the logistic regression model pointed out an odds ratio of 38.5 (95% CI: 9.9–150.6; *p* < 0.0001) to have MVP for subjects with  *p*(*Y*_*i*_ = “MVP”)  > 0.5.

### 3.4. Combination of Osteoprotegerin and Oxidative Stress as Potential Circulating Marker of MVP

**Figure 3:**
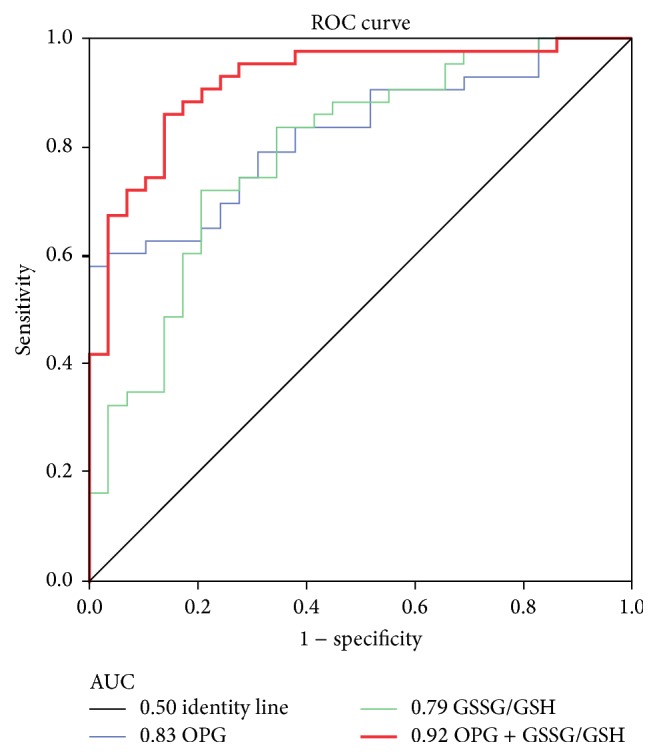
Osteoprotegerin combined with oxidative stress, as potential circulating marker of MVP. Receiver operating characteristic (ROC) curves and area under the curve (AUC) of Osteoprotegerin (OPG), ratio between oxidized (GSSG) and reduced (GSH) form of glutathione (GSSG/GSH) and their combination (OPG + GSSG/GSH). (Control subjects *n* = 29, Mitral Valve Prolapse patients *n* = 43; *p* < 0.0001).

Receiver operator characteristic curve (ROC) was performed to determine if OPG or GSSG/GSH ratio alone or together could be used to identify MVP patients. As shown in Figure 3, OPG and GSSG/GSH ratio had an area under ROC curve (AUC) of 0.83 and 0.79, respectively (*p* < 0.0001). However, if we considered OPG combined with GSSG/GSH we observed accuracy in terms of AUC of 0.92 with 95% CI: 0.86–0.98 and *p* < 0.0001 (red thick line; Figure 3).

## 4. Discussion

Myxomatous mitral valve prolapse (MVP) with severe regurgitation (MR) is the most common cause for mitral valve surgery. The MVP diagnosis is some of the most challenging aspects in clinical cardiology and, up to now, echocardiography is the only clinical reliable tool. No biomarkers are available for this pathology and, to the best of our knowledge, only a comparative proteomic study on plasma samples from 24 pooled MVP patients with moderate to severe MR revealed reduced levels of haptoglobin, platelet basic protein, and complement component C4b in the MVP/MR patients as compared to the 24 pooled matched control cases [16]. However, the clinical applicability is unclear, in part because most of the identified biomarkers had low AUC. In addition, Thalji et al. [12] and Sainger et al. [17] identified novel players that could be involved in myxomatous mitral valve degeneration and their data offer novel insights into the pathogenesis of MVP but no circulating biomarkers have been evaluated.

Results of the present work show that circulating OPG and oxidative stress status are positively associated with severe MR due to MVP. Based on these premises, we developed a multivariable logistic regression model with OPG and GSSG/GSH levels. This model is able to correctly identify 86% of MVP patients and 86% of control subjects.

Since all patients, in the MVP group, were the ones who underwent surgery due to symptoms, we evaluated and excluded that left ventricular ejection fraction (LVEF) and heart failure (NYHA classes) were the causes that increased OPG plasma levels and oxidative stress status. Therefore, our data supports that severe MR caused by myxomatous MVP could be identify through a multivariable binary logistic regression model.

The analyses of OPG as well as oxidative stress alone are not disease specific; indeed OPG has already been linked to coronary artery syndromes [18], aortic valve stenosis [19], myocardial infarction [20], and cardiovascular postoperative outcome [21]. However, the combination of these biochemical parameters allowed us to identify these patients with high accuracy.

Recently, we showed that OPG was involved in mitral valve endothelial to mesenchymal transition and plasma OPG levels permitted to identify MVP patients with an AUC of 0.92 [8]. However, all the selected MVP patients had only posterior MVP. It is known that two-thirds of MVP patients present the posterior prolapse, while the remaining third show anterior or bileaflet prolapse [22, 23]. Considering this, to obtain a model able to discriminate MVP from healthy subjects, in primary screening, we must use a population with not only posterior but also anterior and bileaflet prolapse. Thus, in the present study we enrolled 43 MVP patients: 28 with posterior MVP (65%) and 15 with anterior or bileaflet MVP (35%). In this population, OPG had an AUC of 0.83 that was brought back to 0.92 when OPG was combined with GSSG/GSH ratio; meaning that OPG alone is not sufficient to discriminate with high accuracy the MVP mixed population (posterior, anterior, and bileaflet) from healthy subjects.

The present study has some limitations: first, the exiguous number of patients enrolled and the lack of strong disease-specificity of OPG and oxidative stress with MVP. In addition, in our cohorts we had only two cases with mitral annulus calcification (MAC). Since OPG is involved in many cellular processes, in particular calcification, further studies need to address also the possible implication of this molecule not only in patients with MAC but also in those who have mitral valve stenosis. Lastly, patients with severe regurgitation compose the MVP cohort and further studies are needed to evaluate if this model is able to discriminate mild or moderate mitral valve regurgitation in the presence or absence of prolapse.

## 5. Conclusion

In conclusion, considering the prevalence of this disease (over 6% ≥65 years old [1]), the aging population, and the price of the echocardiographic evaluation, the health system costs may rise if no pharmacological treatment or cheaper diagnosis tools will be identified. In particular, circulating molecular signatures could be used in primary screening to identify possible MVP patients and then a confirmatory echocardiography could be performed only in these subjects. Thus, the study of new circulating markers and the combination of already known ones involved in MVP progression could lead to novel insights and possibly new therapeutic targets. To the best of our knowledge, this is the first study to show a strong association between OPG and oxidative stress status. In addition, these molecules could be measured in the clinical setting by the implementation and validation of diagnostic enzyme-linked immunosorbent assay (ELISA) for OPG and colorimetric assay for GSH and GSSG. Finally, since it is quite hard to believe that one single protein could discriminate two populations with high specificity and sensitivity, we believe that this approach could improve the identification of several signatures not only in mitral valve disease. However, the mechanisms explaining these correlations are still unclear; further molecular studies along with clinical validations will be necessary to confirm our findings.

## Figures and Tables

**Table 1:** Patient demographics.

	Control (*N* = 29)	MVP (*N* = 43)	*p* value
*Variable*			
Age (years)	57.3 [53.2,61.4]	60.1 [56.8,63.5]	0.291
Sex (male)	19 (65.5%)	29 (67.4%)	0.865
Diabetes	3 (10.3%)	3 (7.0%)	0.685
Hypertension	14 (48.3%)	16 (37.2%)	0.660
Hypercholesterolemia	14 (48.3%)	25 (58.1%)	0.688
Smokers	6 (20.7%)	5 (11.6%)	0.514
BMI	27.4 ± 0.76	24.8 ± 0.43	**0.005**
Total cholesterol (mg/dL)	216.1 ± 7.4	215.2 ± 6.6	0.973
Triglycerides (mg/dL)	111.2 ± 9.0	110.3 ± 6.7	0.690
HDL (mg/dL)	55.8 ± 3.2	51.3 ± 1.9	0.249
LDL (mg/dL)	138.0 ± 7.1	132.4 ± 6.5	0.658
NYHA class			**0.001**
I	29 (100%)	19 (44.2%)	
II	—	17 (39.5%)	
III	—	7 (16.3)	
IV	—	—	
*Drug therapies*			
Antiplatelets (%)	1 (3%)	2 (5%)	1.00
Angiotensin receptor blockers (%)	4 (14%)	3 (7%)	0.429
Converting enzyme inhibitors (%)	5 (17%)	14 (32%)	0.295
Calcium channel blockers (%)	3 (10%)	3 (7%)	0.679
Beta-blockers (%)	1 (3%)	16 (37%)	**0.007**
Nitrates (%)	0 (0%)	1 (2%)	1.00
Statins (%)	6 (21%)	6 (14%)	0.527

BMI: body mass index; HDL: high-density lipoprotein; LDL: low-density lipoprotein; NYHA: New York Heart Association. [minimum, maximum]; (percentage); mean ± standard error.

**Table 2:** Echocardiography evaluation.

Echocardiography parameters	Control (*N* = 29)	MVP (*N* = 43)	*p* value
LVEF (%)	65.9 ± 1.6	63 ± 1.6	0.416
Peak *E* velocity (cm/s)	72.8 ± 3.8	103 ± 4.2	**<0.001**
Deceleration *E* (ms)	229 ± 13.4	199 ± 7.7	0.094
Peak *A* velocity (cm/s)	79.1 ± 4.8	68.9 ± 2.8	0.111
*E*/*A* ratio	0.96 ± 0.07	1.6 ± 0.1	**<0.001**
EROA (cm^2^)	—	0.6 ± 0.04	

LVEF: left ventricular ejection fraction; EROA: effective regurgitant orifice area. Mean ± standard error.

## Abbreviations

AUC: Area under the curve
BMI: Body-mass index
GSH: Reduced glutathione
GSSG: Oxidized glutathione
HDL: High-density lipoproteins
MAC: Mitral annulus calcification
MVP: Mitral valve prolapse
OPG: Osteoprotegerin.
