# Corrigendum to “Identification of Patients Affected by Mitral Valve Prolapse with Severe Regurgitation: A Multivariable Regression Model”

**DOI:** 10.1155/2017/3575348

**Published:** 2017-09-24

**Authors:** Paola Songia, Benedetta Porro, Mattia Chiesa, Veronika Myasoedova, Francesco Alamanni, Elena Tremoli, Paolo Poggio

**Affiliations:** ^1^Centro Cardiologico Monzino IRCCS, Milan, Italy; ^2^Dipartimento di Scienze Farmacologiche e Biomolecolari, Università degli Studi di Milano, Milan, Italy; ^3^Dipartimento di Scienze Cliniche e di Comunità, Università degli Studi di Milano, Milan, Italy

In the article titled “Identification of Patients Affected by Mitral Valve Prolapse with Severe Regurgitation: A Multivariable Regression Model” [1], references [2, 3] should have been included as references 24 and 25 in the original article.

Accordingly, in the introduction section, the text reading “Echocardiographically, MVP is defined as a single or bileaflet prolapse, at least 2 mm beyond the long-axis annular plane, while the assessment of valve regurgitation takes into account the effective regurgitant orifice area (EROA) [2]” should be changed to “Echocardiographically, MVP is defined as a single or bileaflet prolapse, at least 2 mm beyond the long-axis annular plane, while the assessment of the severity of MR relies on several parameters according to the current recommendations [24, 25].”

Additionally, the Acknowledgments section should be updated as follows:

“The authors thank the entire Cardiac Surgery Unit at Centro Cardiologico Monzino for the clinical data and blood collection. The authors also would like to thank Dr. Paola Gripari for patient evaluation and critical review. This work was supported by the Fondazione Gigi e Pupa Ferrari ONLUS and the Italian Ministry of Health [RC2015-BIO30-2613051 to Centro CardiologicoMonzino].”
